# Discourses on children and health in National Public Health Policy documents: examples from Sweden and Norway

**DOI:** 10.1093/heapro/daag073

**Published:** 2026-06-09

**Authors:** Ulrika Lögdberg, Zofia Hammerin, Gita Hedin, Olin Oldeide, Thomas Tjelta, Berit Misund Dahl, Anne Clancy

**Affiliations:** Karolinska Institutet, Department of Learning, Informatics, Management and Ethics (LIME), National Centre for Suicide Research and Prevention (NASP), 171 77 Stockholm, Sweden; Department of Education and Art, Mälardalen University, Universitetsplatsen 1, 721 23 Västerås, Sweden; Faculty of Health and Science, Kristianstad University, Elmetorpsvägen 8, 291 88 Kristianstad, Sweden; Department of Health Promotion and Development (HEMIL), University of Bergen, P.O. Box 7807, NO-5020 Bergen, Norway; Faculty of Health and Social Sciences, University of South-Eastern Norway, Kjølnes Ring 56, 3918 Porsgrunn, Norway; Department of Health Sciences, NTNU-Norwegian University of Science and Technology, P.O. Box 1517, NO-6025 Ålesund, Norway; Faculty of Health Sciences, Department of Health and Care Sciences, UiT, the Arctic University of Norway, Postboks 1063, 9480 Harstad, Norway

**Keywords:** agency, children, critical discourse analysis, health promotion, policy, young people

## Abstract

Promoting the health of children remains a global and national priority, as reflected in the proliferation of public health policies, strategies, and guidelines over the past decade. While these documents aim to improve health outcomes, they also play a critical role in shaping how children and young people are constructed as health subjects. This study critically examines recent national public health policy documents from Sweden and Norway through a health promotion lens, focusing on the discursive positioning of children. Drawing on theoretical insights from critical childhood studies and public health policy research to frame our discourse analysis, we identify dominant narratives that frame children primarily as passive, dependent, and vulnerable. These adult-centric discourses, rooted in risk and knowledge paradigms, offer limited space for agency, participation, or rights-based approaches. Despite strong rhetorical commitments to equality and child health, the policies risk reinforcing hierarchical power relations and deficit-based views of childhood. We argue that aligning public health policy with health promotion principles requires reframing children not merely as future investments, but as present rights-holders and active contributors to their own health. Embedding participatory mechanisms and expanding comparative research across welfare contexts may foster more inclusive and empowering policy development. This study contributes to ongoing debates on child agency, policy framing, and the role of discourse in shaping health promotion practice.

Contribution to Health Promotion

- Highlights how public health policies shape views of children and young people.
- Shows that Nordic policies often portray children as passive and vulnerable.
- Calls for more child participation in health policy design and evaluation.
- Suggests reframing children as active contributors to their own health.
- Supports policy change towards empowerment and rights-based approaches.

## Introduction

Promoting the health of children and young people is a stated priority in global and national policy arenas. International frameworks such as the Global Strategy for Women’s, Children’s and Adolescents’ Health 2016–2030 (EWE Child 2015) articulate broad commitments to equity, participation and improved health outcomes. At the national level, countries such as Sweden and Norway regularly update policies and guidelines concerning child health. A recent example is the publication of national recommendations on screen use (Nordic Welfare Centre 2025). More broadly, the past decade has witnessed a marked increase in the production of health-related policy documents, strategies, and guidelines, both internationally and domestically (Paichadze *et al*. 2025). This expansion reflects the notion of policy as a key instrument for shaping health outcomes in practice, not only by setting priorities and mobilizing resources but also by influencing everyday health behaviours and the environments in which people live. Yet policy representations are never neutral: they construct particular understandings of, in this context, childhood, health, and risk and thereby delimit which problems are identified and which solutions are considered legitimate (Fischer and Forester 1993, de Graaff *et al*. 2025).

From a health promotion perspective, which emphasizes empowerment and participation, it is therefore important to scrutinize how policy texts construct children and young people as health subjects. The Nordic context offers a particularly relevant setting for analysis. Sweden and Norway are internationally recognized for their strong welfare states and for prioritizing children’s rights and participation (e.g. SIDA 2000, Haugli *et al*. 2020). At the same time, policy framings in these contexts are not immune to political currents, normative assumptions, and competing discourses. Examining how children and young people are described in recent public health policy documents from these two countries may therefore shed light on the tensions between ideals of participation and empowerment on the one hand and policy framings that may constrain agency on the other. The aim of this study is thus to analyse the discourses surrounding children and young people and health as presented in recent national public health policy documents from Sweden and Norway within a health promotion framework. The study addresses the following research questions:

1. What are the dominant discourses regarding children and young people and health in the two policy documents?
2. How are the children and young people positioned within these discourses?

### Previous research

A growing body of research shows that children’s perspectives remain marginal in global health and policy contexts. For example, studies demonstrate that children under 15 years are often underrepresented in global health research (Hunleth *et al*. 2022), despite their unique experiences, priorities, and right to participate (Tisdall and Cuevas-Parra 2022), with ethical and methodological challenges often limiting participation (Wademan *et al*. 2024). Excluding children’s voices risks producing policies and interventions that fail to reflect lived realities and constrain children’s agency in both national and global knowledge production (Wademan *et al*. 2024).

When children are included in global health research, their participation often remains limited unless research designs actively address adult–child power asymmetries (Lundy 2018, Spyrou 2018, Hunleth *et al*. 2022). This is the case despite studies undertaken in diverse global contexts demonstrating that young children can self-report on issues concerning their health (Hietanen *et al*. 2025) articulating nuanced understandings, e.g. of treatment experiences, vaccination practices, caregiving responsibilities, and health priorities when participatory, dialogical, and creative methodologies are employed (Christensen and James 2017).

Rather than treating knowledge as something extracted from children, recent scholarship conceptualizes knowledge as coproduced through interaction (Christensen and James 2017, Spyrou 2018). This work challenges the dominant positioning of children as passive objects of protection and instead frames children as situated knowledge holders whose perspectives can meaningfully inform health policy and programme design. A recent systematic review shows that children value their rights as citizens and the fulfilment of those rights (Fairhall and Woods 2021). This suggests that children’s marginalization in policy processes is not primarily a matter of capacity, but of epistemological assumptions embedded in research and governance structures.

Similar patterns emerge in policy analysis, where World Health Organization texts frame equity as discursively constructed to prioritize adult-defined concerns (Amri *et al*. 2023). Together, these findings suggest that children’s exclusion is not a mere methodological oversight, but a product of broader policy framings, rendering children’s experiences peripheral despite stated commitments to equity and participation. Systematically integrating children’s perspectives is therefore crucial for more inclusive and ethically robust global health policies (Wademan *et al*. 2024).

Questions concerning children’s place in policymaking further underscore these tensions. Although children have become more visible in policy rhetoric, their participation is often symbolic or highly controlled by adults (Stenvall *et al*. 2023), a dynamic echoed in child protection research in which culturally embedded discourses position children as vulnerable, lacking competence, or insufficiently reliable to influence assessments (Jørgensen *et al*. 2025). This convergence across fields reveals how rights-based frameworks do not necessarily translate into meaningful influence, as discourses of protection and vulnerability continue to justify limiting the space available for children’s voices. At the same time, studies of school-based health promotion highlight similar barriers: while institutions increasingly emphasize student engagement, hierarchies and adult gatekeeping persist, limiting the degree to which children and young people can shape health initiatives on their own terms (Kontak *et al*. 2025). Together, these studies illustrate how deeply rooted assumptions about children’s capacities shape whether their perspectives are considered in different sectors.

These patterns are particularly visible when examining the broader structural forces shaping the health of children and young people. A growing strand of work on commercial determinants of health shows how children and young people are targeted by industries, such as food, alcohol, and digital media, with marketing strategies directly influencing health outcomes (Pitt *et al*. 2024, Francis *et al*. 2025). While these studies point to the need for stronger regulatory and advocacy responses, they also challenge the dominant protectionist model that frames children and young people primarily as passive victims. Instead, they call for approaches that recognize children and young people as rights-bearing actors capable of navigating and resisting commercial pressures. This critique resonates with longer-standing debates in childhood studies on whether children are conceptualized primarily as ‘human becomings’ oriented towards future citizenship, or ‘human beings’ with present-tense agency (James 1998, Uprichard 2008, Peleg 2019). Research on mental health interventions shows how the ‘becoming’ frame continues to dominate, positioning children and young people as shaped by adult influences and responsible for individual behavioural change, rather than as situated within social and structural conditions (Bergnehr and Zetterqvist Nelson 2015, Bergnehr and Gunnarsson 2025). Researchers further challenge the notion of a singular, authentic ‘child voice’, arguing instead for real understandings shaped by context and interaction, an insight with important implications for how participation is conceptualized in policy, e.g. (Facca *et al*. 2020).

While many studies highlight structural and discursive barriers to meaningful participation, research also illustrates that children’s involvement can substantially strengthen health and welfare policies when appropriate mechanisms are in place. For instance, although school-based health promotion initiatives often remain constrained by institutional hierarchies and adult gatekeeping (Kontak *et al*. 2025), other models demonstrate what more meaningful engagement can look like. The Children in All Policies 2030 initiative offers one such example: a Youth Advisory Board of young people from 17 countries contributed across all stages of policy development, generating insights that improved both the relevance and quality of global health decision-making (Mathews *et al*. 2025). These findings resonate with wider critiques that call for moving beyond symbolic participation (Stenvall *et al*. 2023) by creating structures that recognize children not merely as consultees but as knowledgeable actors whose perspectives can shape policy in substantive ways. Taken together, this research suggests that the barriers to child participation are not inherent to children themselves but stem from the design of policy processes and that when these structures are reconfigured children’s contributions demonstrably enhance policy and programme outcomes.

To summarize, this body of research demonstrates that children and young people’s involvement in health policy cannot be reduced to questions of whether they are present, consulted, or represented. Instead, a critical issue is how they are discursively positioned within policy texts and research practices, and how these positions shape the range of possible interventions and the legitimacy granted to the perspectives of children and young people. Policy language plays a central role in constructing childhood and health across fields, determining which priorities become thinkable and which forms of agency are recognized. Understanding these discursive dynamics is therefore essential for developing more equitable, participatory, and contextually grounded approaches to child health and well-being.

Against this backdrop, the present study draws on theoretical insights from critical childhood studies and public health policy research to frame our discourse analysis. While health promotion literature often addresses children and young people as targets of intervention, it has historically been shaped by biomedical and behavioural paradigms that tend to foreground risk reduction, individual responsibility, and expert-led prevention (Lundy 2018, Kett *et al*. 2025). Such framings are rooted in broader historical and disciplinary separations between social science perspectives on childhood and mainstream health and psychological science. Critical childhood studies have long challenged these tendencies by emphasizing childhood as a socially and historically constructed category and by questioning developmental and deficit-oriented models of children’s well-being (James and Prout 1997, Mayall 2002, Spyrou 2018). Building on this tradition, the present analysis contributes to ongoing debates by examining how children are discursively positioned within health policy texts. In doing so, the study responds to calls within childhood studies to more explicitly engage with health promotion fields and extend critical analyses of power, agency, and subjectification into policy-oriented public health research (e.g. Mayall 2002, Burman 1994/2017, Spyrou 2018).

## Materials and methods

This study employs critical discourse analysis (CDA) grounded in Fairclough’s three-dimensional framework (Fairclough 1992, 2010). CDA views language as a form of social practice through which meanings, identities, and power relations are produced and reproduced. From this perspective, policy texts are understood as sites where understandings of childhood and health are constructed and negotiated. The approach is grounded in social constructivist epistemology, which emphasizes that knowledge and social categories are historically and culturally situated rather than fixed or neutral (Burr and Dick 2017).

The analysis is informed by perspectives from critical childhood and youth studies, where childhood is understood as socially constructed rather than solely defined by developmental stages. Within this field, scholars have long debated whether children are primarily conceptualized as ‘human becomings’, future adults in the process of development, or as ‘human beings’ whose present experiences and perspectives hold intrinsic value (James 1998, Uprichard 2008). These conceptual tensions provide a backdrop for examining how policy discourse positions children in relation to health, agency, and responsibility.

Relatedly, ideas concerning adult authority and social power form part of the broader interpretive framework of the study. Adultism, understood as the systemic privileging of adult knowledge, authority, and decision-making power over children and young people (Bell 1995; Flasher 1978), is considered here as a contextual concept informing critical interpretation rather than as a primary analytical lens.

Drawing on Foucauldian perspectives on power as productive (Foucault 1979, Fairclough 1992), policy discourse is viewed as contributing to the formation of subjectivities by shaping normative expectations regarding agency, responsibility, and risk. In this sense, policy language may function as a governing mechanism that influences how children and young people are imagined within institutional and social structures, even when participation is formally acknowledged.

The study also draws on positioning theory (Harré and Moghaddam 2003, van Langenhove 2021) to examine how policy language locates children and young people within particular roles and expectations. Positioning theory enables analysis of how texts attribute rights, responsibilities, and forms of agency, e.g. constructing children as vulnerable populations, future citizens, dependents, or contributors to knowledge. In line with critical perspectives in childhood studies (Burman 1994/2017, Hunleth *et al*. 2022), the analysis assumes that such discursive positioning plays an important role in shaping broader social understandings of childhood.

### Material

The material for analysis comprised two national public health policy documents: one from Sweden (The Public Health Agency of Sweden 2020) and one from Norway (Government.no 2023), selected for their status as central governmental strategies in effect during 2023–2024. The documents present national strategies to reduce health inequalities and describe support structure for coordinated public health initiatives. These documents articulate official positions and priorities regarding public health and are understood as political texts that shape discourse, guide action, and influence how health and childhood are constructed in society (Fairclough 2010).

*På väg mot en god och jämlik*  *hälsa* (2020), issued by the Public Health Agency of Sweden, provides a comprehensive structure for coordinating national public health efforts, emphasizing systematic, cross-sectoral collaboration and highlighting shared responsibility for creating the conditions for good and equitable health. The document addresses a broad audience, including professionals, civil society, and the general public, and is organized around eight national target areas, ranging from early life conditions and education to lifestyle habits and equitable healthcare. *Folkehelsemeldinga* (2023) issued by the Ministry of Health and Care Services following a debate in the Norwegian Storting functions more explicitly as a strategy aiming to reduce health inequalities through clear national priorities. Aimed at both professionals and the wider public, this focuses on six strategic areas, including mental health, prevention of noncommunicable diseases, citizen engagement, and environmental health.

From a discourse analytical perspective, these texts contribute to meaning-making processes and reflect ideological framings. Examining how children are positioned within these documents provides insight into the values, assumptions, and power relations embedded in health policy. While neither document focuses specifically on children, both reference children in relation to broader societal structures. The analysed data consists of publicly available documents; therefore, the article has not been sent for ethical approval to relevant authorities.

### Data analysis

We followed Fairclough’s (2010) method, which includes three steps. The first step involved taking a linguistic approach. The Norwegian and Swedish authors analysed their respective countries’ documents individually before sharing, comparing, and contrasting their interpretations firstly within and secondly between groups. The readings of the documents focused on sections referencing children and youth. This included analysing word choices, the argumentative structure of the text, the strength of statements, and the conceptual positioning of children. Key aspects included whether children were depicted as active, passive, empowered, or in need of protection. NVivo v13 (Lumivero 2020) was used in this first step to generate abridged versions of the texts using word queries based on keywords, such as child, young, school, student, grade, and class. These search terms were selected to capture the broader context of references to children and young people. Additional queries were conducted to ensure a thorough analysis of terms relevant to the health of children and young people, including health promotion and prevention. This approach enabled the identification and exploration of specific passages within the documents that construct children in relation to health, providing a comprehensive basis for critical discourse analysis.

In the second step, we examined intertextuality, including links to other documents and the interplay of genres and styles of discourses. The analysis explored how the discursive positioning of children was articulated and sought to uncover hidden ideologies embedded in the text. These ideologies were analysed in relation to discursive power structures and the concept of hegemony. The broader sociopolitical context was analysed in the final step to identify dominant ideologies and hidden agendas within the documents. The focus was on whether the documents reflected participation ideologies, addressed equality, and supported children’s agency over their health and well-being. The relationship between the text, discursive practice, and social practice is elucidated in the discussion. The analytical process described above was conducted in Swedish and Norwegian and translated into English. Recurring meetings and discussions ensured consistency in the analysis. To present the findings, illustrative quotes were selected from each identified discourse to highlight patterns, including recurring common and divergent themes within the documents.

## Results

### Findings from the Swedish policy document

In the Swedish document, three distinct discourses were identified: two dominant discourses, (i) the ‘dependency discourse’ and (ii) the ‘control discourse’, and a third, less dominant discourse, (iii) ‘being listened to’.

### Dependency discourse

The dependency discourse focuses on the dependency of children and young people on adults in order to be healthy. Their learning environment, home environment, and play are described as in need of adult management. Children and young people are positioned as passive as opposed to active agents in their own lives and health. The first example of this discourse is from a section of the document called ‘An equitable preschool of high quality’, which is one of several key areas for health, mentioned in the document:

Besides educational activities and care, play is important for children’s social, cognitive, and motor development. For this, an environment that stimulates conversation, play, and physical activity, both indoors and outdoors, is needed. (p. 39)

The emphasis on structured play reflects a societal expectation that children need adult oversight and intervention and the importance of a stimulating environment, structured by adults to enable appropriate development. By highlighting children’s development in specific domains (social, cognitive, motor) and underscoring the need for particular settings, the text implies that children do not naturally achieve these skills. This frames children as being in need of adult intervention in their play.

The excerpt lacks the children’s own perspectives, experiences, and choices regarding play. It does not explore which types of play or activities are encouraged in different environments, nor does it consider how cultural and socioeconomic factors may influence access to these resources. Here, ‘play’ is defined in terms of its developmental benefits rather than as a self-directed activity, which narrows the view of play as a structured tool for development rather than a free and creative activity.

Another example that describes children as dependent:

To achieve good and equitable health, it is important to create, support, and strengthen a good start in life and equitable conditions for growing up so that all children have the fundamental opportunities to develop cognitive, emotional, social, and physical abilities based on their individual circumstances. (p. 38)

Words such as ‘good and equitable health’, ‘good start’, and ‘equitable conditions’ suggest a normative view of children’s lives that prioritizes equity and well-being but also subtly positions children as dependents. This passive positioning reinforces children’s lack of autonomy, suggesting they rely on adults to ‘create, support, and strengthen’ foundational conditions for them.

A final example of the dependency discourse from a section called ‘Income and means of support’ is illustrated in the following quote:

Growing up with limited financial resources can negatively impact children’s lives in several ways, such as through less healthy lifestyles, poorer academic performance, and overcrowded living conditions. (p. 59)

The excerpt does not indicate who or what is responsible for creating and sustaining these financial limitations. Children are positioned as passive victims of financial hardship. The statement does not acknowledge how children and families navigate or resist these challenges, reinforcing a deficit-based view of childhood poverty. The excerpt does not include children’s perspectives on how they might navigate economic challenges. By focusing on ‘less healthy lifestyles’ and ‘poorer academic performance’, the discourse risks reinforcing stereotypes about disadvantaged children and diverts attention from the need for structural change, such as labour policies, housing affordability, and education funding, which play a critical role in childhood poverty.

### Control discourse

In this discourse, the focus is on children being at risk and needing adult protection and control to avoid ill health. Different risks are mentioned, e.g. overcrowding, eviction, crime, inadequate housing, poor diet, and a sedentary lifestyle. Children are primarily positioned as at risk from various dangers and not as strong, healthy, and capable individuals. The first example from this discourse is from the section entitled ‘Competence, knowledge and education’:

It is possible to improve a child’s learning opportunities by early identification of children with learning difficulties and providing support and adaptations to meet their needs. It is also important to identify primary school students with high absenteeism at an early stage. (p. 46)

The use of ‘providing support and adaptations to meet their needs’ reinforces an adult-controlled model of care, where agency is predominantly held by adults and institutions. The structure of the sentence assigns responsibility to adults for ‘identifying’, ‘providing support’, and ‘adapting’ the system, while children are positioned as recipients. Nor is there a mention of children influencing the learning environment. This can be seen as contributing to an adult-centred discourse where children are in need of protection and safety provided by adults. The absence of child agency in shaping their own learning experiences or explaining their own absenteeism reinforces a hierarchical power dynamic where adults diagnose, assess, and intervene.

Another example of the passive portrayal of children is evident on page 63, where Sweden’s public health goals are described. One of the goals concerns children:

To strengthen the ability and opportunity for social participation for people in economically and socially vulnerable situations, and to enhance the protection of vulnerable children. (p. 63)

The wording ‘to strengthen the protection of vulnerable children’ constructs children primarily as in need of safeguarding, rather than as rights-holders. The word ‘protection’ implies a passive role for children, with adults or institutions acting as their protectors. The sentence lacks an explicit responsible agent. This passive construction removes responsibility from specific institutions (e.g. government, schools, and welfare systems), making it unclear who holds power to enact these changes. The discourse does not acknowledge children’s agency in influencing their social participation, health, or protection.

The excerpt reflects a discourse prevalent in educational and policy texts that prioritize structured, adult-managed environments as essential for child development. This discursive practice normalizes a view where children are positioned as developing entities rather than as autonomous agents.

### Being listened to

Occasionally, a less dominant discourse is detected. In a section in the document on the interplay of various key areas, a sentence regarding children stands out:

It is about ensuring that children are treated with respect and listened to, which contributes to the development of participation and influence. (p. 41)

The emphasis in the excerpt is on respect and being listened to. However, no examples of participation or influence are mentioned. The excerpt introduces the idea that children should be ‘treated with respect and listened to’, which seems to signal a shift towards empowering children by valuing their perspectives. However, while this positioning may seem empowering, being listened to does not guarantee meaningful participation or equal influence in matters of importance to them. This suggests that respect and listening are means to an end (developing influence), rather than inherently acknowledging children’s voices and agency. The formulation implies that respect is something given to children rather than a mutual expectation, maintaining an implicit adult authority over granting or withholding that respect.

Another excerpt is from a section called ‘Health promoting healthcare meetings’:

There is also a need for information on children’s experiences of healthcare encounters, which is currently lacking. (p. 86)

The phrase ‘a need for information’ frames children’s experiences as something that must be collected and studied rather than inherently valued or actively integrated into healthcare practices. ‘Children’s experiences of healthcare encounters’ implies that children’s perspectives are important, but their role in shaping healthcare practices and decision-making remains unspecified. The focus is on gathering information rather than centring children’s voices in healthcare structures.

The phrase ‘which is currently lacking’ highlights an absence of knowledge but does not identify why this information is missing or who is responsible for ensuring children’s perspectives are considered. Children are positioned as objects of study rather than as active participants in shaping healthcare. The discourse focuses on the need to ‘gather information’ rather than empowering children to influence healthcare policies and practices.

### Findings from the Norwegian policy document

Three discourses were identified in the Norwegian public health document: two dominant discourses, (i) a ‘risk discourse’ and (ii) a ‘knowledge discourse’, and a third, less dominant, (iii) ‘participation discourse’.

### Risk discourse

Throughout the Norwegian document, children are portrayed as needing protection both from the state and responsible adults, such as teachers and health and social care professionals. This positions children as passive recipients of support, rather than coconstructors of their own development. The agency lies entirely with adults, who are presented as central actors in shaping children’s futures. Their goal is to protect children and young people from a broad range of health risks, including sedentary behaviour and the negative effects of digital media and substance use, frequently framed in the context of social inequities as in the following text excerpts:

There is cause for concern regarding both the generally low levels of physical activity among children and young people, attrition from organized sports activities, and barriers to participation still exist. It is imperative that all children and young people are able to participate, regardless of gender and the socioeconomic status of their parents. (p. 57)

Many young people risk exposure to negative experiences online. Many may also be exposed to hazardous online content: violence and aggression, suicide, and self-harm. Vulnerable young children, in particular, may be at greater risk from hazardous user-generated online content. (p. 97)

These sections emphasize children and young people’s ‘exposure’ to risks associated, e.g. with low levels of physical activity and their engagement with online content, including advertising, pornography, and graphic violence, while highlighting the disproportionate impact on vulnerable groups. Children and young people are constructed as passive both in terms of the socioeconomic status of their parents and their ‘exposure’ to online harms: they ‘risk exposure’ rather than engaging with or navigating digital content. While there are some references to the benefits of digital environments for children and young people, the excerpts are explicitly focused on risk and protection.

The section continues to highlight that:

Children are particularly vulnerable to marketing pressure and shall be given special and effective protection. (p. 98)

Here, the modal auxiliary verb ‘shall’ is used, stating that children shall be protected from marketing pressure, which shows statement strength. The repeated use of ‘risk’, ‘exposure’, and ‘hazardous content’ constructs the online world as a threatening and dangerous space, particularly for children. ‘Vulnerable young children in particular’ reinforces the idea that certain groups of children are especially at risk, which may imply a generalized view of vulnerability rather than a nuanced, context-based understanding.

The main theme in the document is to reduce social inequities in health, related to children. A recurring theme is to protect children from the effects of social inequity. It is described as a problem that some urban areas have a high proportion of children from poor families. Young people are described as being at risk of ‘falling’ out of education, and the necessity of identifying and ‘catching them’ before they ‘fall’ is specified in the document:

It is therefore especially important to identify and catch young people who are at risk of falling outside education or employment early so that they will get necessary support to complete their education and participate in working life. (p. 32)

The document illustrates that a risk discourse is produced with the overarching goal of protecting children from a range of specified threats. Although the language is unambiguous, children ‘should be protected’, how these aims are to be realized is not always specified in the text. These risks can be ameliorated through reduced inequalities, but there is little focus on children as active agents.

### Knowledge discourse

In this discourse, competent adults, such as parents and professionals, are mentioned. A section of the report frames outcomes as ‘determined’ by parental education. Health and social care professionals, teachers, and other staff play an essential part in constructing safe environments for children. Competency is seen as a key instrument in doing so. Completing secondary school is described as the foundation for good health later in life. It is further purported that knowledge and competencies are essential in reducing social inequities.

The competence of teachers and other staff is crucial for preventing problems and supporting all children and pupils so that they thrive, feel confident, and flourish. (p. 28)

The term ‘crucial’ underscores the importance of competency, but strong modal verbs, such as should and must that express a strong sense of necessity, urgency, or obligation, are not used. The term ‘crucial’ suggests a strong dependency on adult expertise, implying that children’s ability to thrive is contingent on professional intervention. The phrase ‘preventing problems’ implies that children are potential sites of risk, subtly framing them as vulnerable or prone to issues unless effectively managed by competent adults. ‘Supporting all children and pupils’ is inclusive in tone, but ‘supporting’ again reinforces a top-down adult-to-child relationship, positioning children as dependent on external help.

The terms ‘thrive’, ‘master’ their lives, and ‘flourish’ are strongly positive and aspirational. However, they are also vague and idealistic, leaving open the question of who defines what it means to ‘flourish’ or ‘master’ life and by what criteria. Children are not active agents in this construction; their thriving and flourishing are dependent outcomes, contingent on adult competence.

The knowledge discourse is re-produced throughout the document, where knowledge and competencies are described as essential in addressing inequities. This is linked with the prevention discourse where adults have the responsibility of protecting children from ill-health. Although the report also highlights positive aspects of digitalization and opportunities in this context, the focus on prevention can indicate that the dominant knowledge discourse refers to expert knowledge. The text implies that the problem can be fixed and that knowledge is the answer, implicitly through education. Knowledge is based on expert advice from professionals and other competent adults, aligning with the broader discourse on competencies where increased knowledge equates to greater protection without specifying how this protection is achieved. Poor families are mentioned as a particularly vulnerable group where expert knowledge is seen as a solution to alleviate their challenges.

The government has appointed an expert group to examine children in poor families and an expert group that will examine the impact of kindergartens, schools, and after-school care on social mobility. The recommendations from the expert groups will serve as important knowledge bases for future policy documents. (p 26)

The recommendations from experts are deemed ‘an important knowledge base’, signalling a privilege of formal, adult-produced expertise in the construction of policy-relevant knowledge. This positioning marginalizes the perspectives and voices of children and young people.

### A participation discourse

In this discourse, children are assigned a dual role in relation to participation. On the one hand, they are positioned as legal subjects with the right to participate, whereas in a few instances, participation among children is deemed a goal in itself. In the less prominent discourse of participation, two different ways of conceptualizing children’s participation are identified. The following are examples of this dual role:

Children and young people have the right to be heard in matters that concern them, and their opinions shall be given due weight in accordance with their age and maturity. (p. 121)

Participation can in itself be health-promoting by enabling children to become active citizens and feel a sense of belonging in society. (p. 121)

The sentences point to different discourses within participation. The first using the phrase ‘right to be heard’ and ‘opinions shall be given due weight’. This refers to the legal discourse using strong modal verbs to communicate the importance of the written word. The second sentence refers to a discourse framing participation as something that might bring children a sense of value and a process, which could give children influence as citizens. The use of ‘can’ to point at the opportunity of health promotion communicates both that it ‘may happen’ and that ‘it may not happen’. The participation discourse emphasizes that children should actively engage in and make decisions about their own lives. However, this stands in contrast to the risk discourse, which positions children as passive. This authoritative tone is evident in the document on digital media, which excludes children as active agents in decisions about their digital media environment.

Children are positioned as vulnerable, dependent, and passive subjects to be shielded from external threats rather than engaged as media-literate participants. This may be justifiable when considering younger children but applying this as a generalized strategy undermines older children’s evolving competencies and rights to participation. The quote reflects a binary logic common in child protection discourse: children are either protected or endangered, with little space for gradations of competence, resilience, or participation. A summary of the discourses in the Swedish and Norwegian policy documents is presented in Table 1.

**Table 1:** Discourses in the Swedish and Norwegian policy documents.

Theme/discourse	Sweden—positioning of children	Norway—positioning of children	Similarities and differences
Dependency discourse (Sweden)/knowledge discourse (Norway)	Children are positioned as dependent on adult-structured environments. Development is enabled through adult management of play, learning environments, and upbringing conditions.	Children are positioned as dependent on professional expertise. Teachers, professionals, and expert groups are central actors whose competence enables children to thrive and succeed.	**Similarity:** Adult actors hold primary agency in both documents, reflecting adult-centred distributions of authority and expertise. **Difference:** Sweden emphasizes structural environments and conditions for development, while Norway emphasizes professional knowledge and expert competence as key mechanisms.
Control discourse (Sweden)/risk discourse (Norway)	Children are portrayed as vulnerable and in need of protection and control to avoid ill health. Risks are mainly framed in relation to structural conditions, such as poverty, housing, and school absenteeism.	Children are portrayed as exposed to multiple risks, such as sedentary lifestyles, digital media harms, and educational exclusion. Early identification and intervention are emphasized.	**Strong similarity:** Children are constructed primarily as vulnerable and in need of adult protection, legitimizing intervention and control. **Difference:** Sweden focuses more on structural social risks, while Norway highlights behavioural, digital, and educational risks and emphasizes early detection.
Being listened to (Sweden)/participation discourse (Norway)	Participation is framed as children being treated with respect and listened to. However, examples of concrete influence or decision-making are limited.	Participation is framed as a right. Children have the right to be heard, and their opinions should be given due weight according to age and maturity.	**Similarity:** Participation is present but relatively weak compared to protection discourses, with limited influence on decision-making. **Difference:** Sweden frames participation mainly as being listened to, whereas Norway articulates participation more clearly as a legal right and somewhat as a right in itself, although it remains weakly operationalized.

## Discussion

Our findings highlight adult-centric discourses in both Swedish and Norwegian public health policy documents, framing children as vulnerable, dependent, and in need of protection. Although the specific risk emphases differ, both national texts construct children as objects of prevention and control rather than as competent actors in their own health and well-being. From a critical perspective, this can be understood as an expression of adultism, where adult authority and expertise are normalized as the primary basis for decision-making, while children’s perspectives are subordinated. This aligns closely with critiques of child health policy and research, showing how children’s perspectives are systematically excluded from global health agendas (Hunleth *et al*. 2022, Wademan *et al*. 2024) and how child mental health interventions often rely on deficit-based portrayals of passive children (Bergnehr and Zetterqvist Nelson 2015, Bergnehr and Gunnarsson 2025), despite growing evidence of the need for dedicated policy frameworks for child mental health (Atilola 2017) and that children place value on participation (Fairhall and Woods 2021). Our findings suggest that these dynamics extend into Nordic policy texts, which, even while emphasizing equality and rights, reproduce discourses that locate agency and responsibility primarily in adults rather than children themselves.

The dependency discourse in the Swedish document mirrors the knowledge discourse in the Norwegian one, with both positioning parents, teachers, and professionals as the key bearers of expertise. This echoes earlier findings of child protection contexts, where children’s views were often dismissed through professional framings of vulnerability (Jørgensen *et al*. 2025). Comparable discursive patterns appear in other policy domains, e.g. constructions of the ‘included’ and ‘controlled’ student in Norwegian drug prevention discourses reflecting a generalized logic of vulnerability that legitimizes adult control (Halvorsen *et al*. 2025). In this sense, professional knowledge operates not only as support but also as a form of discursive power that defines what counts as valid knowledge and who is authorized to speak. Despite rhetorical commitments to participation, both policy documents marginalize children’s opportunities to influence decision-making. The Swedish document mentions listening to children briefly, and the Norwegian document refers to their right to be heard, yet neither provides any operational mechanisms for participation. This aligns with observations that children’s policy participation is frequently symbolic or adult led (Stenvall *et al*. 2023), as well as research showing that institutional hierarchies and adult gatekeeping restrict children’s influence in school health initiatives (Kontak *et al*. 2025).

The tension between rhetorical recognition and practical marginalization reflects the importance of attending to policy contradictions, where empowerment is invoked in language but constrained in practice (Rappaport 1981). Children are positioned as subjects who may be ‘listened to’, but not as actors who might shape policy content. This positioning illustrates how power operates productively through discourse, shaping the boundaries of possible agency while maintaining adult control over decision-making arenas. These dynamics resonate with the longstanding debate on children as ‘becomings’ rather than ‘beings’ (James 1998, Uprichard 2008, Peleg 2019), a framing that values children for their future potential as healthy adults while sidelining their present rights and agency. At the same time, previous research demonstrates that meaningful participation is possible when structures support it (Mathews *et al*. 2025). Mathews *et al*. (2025) show how young people effectively influenced global health policymaking through the Children in All Policies 2030 Youth Advisory Board, producing insights that strengthened policy relevance and quality. This example highlights that the limitations observed in our material are not inherent to children’s capacities but arise from policy designs that fail to create participatory space. Bringing this contrast into view underscores that Nordic commitments to rights could be operationalized more substantively through participatory mechanisms that acknowledge children as knowledgeable contributors.

From a health promotion perspective, the documents’ reliance on expert-driven solutions risks oversimplifying the complex social determinants of child health. The Norwegian document relies on identifying those at risk and on expert groups to examine poverty and educational inequalities, while the Swedish document emphasizes early identification and intervention strategies. As Amri *et al*. (2023) observe in their critique of WHO texts, such framings delimit which issues and actors become prioritized, often sidelining structural determinants, such as housing, welfare policy, or digital infrastructures. This further reinforces adult-centric governance, where solutions are formulated through institutional expertise rather than in dialogue with children’s lived experiences. Furthermore, this individualizing tendency is also evident in discussions of commercial determinants of health. While Pitt *et al*. (2024) argue for empowerment strategies that support children in navigating harmful commercial influences, the Norwegian policy document frames digital environments primarily as dangers, paying little attention to children’s digital competencies. The emphasis on protection without empowerment risks leaving children less prepared to navigate complex commercial and technological landscapes. Finally, both policy texts privilege professional and expert knowledge at the expense of children’s lived experiences. This is in sharp contrast to Rappaport’s (1981) argument that experts must draw on community knowledge to develop meaningful solutions and to Facca *et al*.’s (2020) call for a relational understanding of children’s perspectives. While recognizing the need to protect children and young people, our findings highlight the need to move beyond tokenistic consultation towards policy development processes that meaningfully integrate children’s insights and everyday experiences.

## Conclusion

### Conclusion and policy implications

Public health policies should embed children’s councils, participatory design processes, or other mechanisms to secure the systematic inclusion of children’s perspectives in evaluations. Such mechanisms may also serve to challenge adult-centric decision-making structures by redistributing voice and influence in policy processes. Further research is needed to explore how children interpret and resist policy framings, building on evidence that discourses both constrain and open spaces for agency. Comparative studies across Nordic and other European contexts could illuminate how different welfare traditions shape the balance between protection and participation.

Reframing children not merely as ‘becomings’ but as ‘beings’, rights-holders with present capacities is required to align policy more closely with health promotion principles and international rights frameworks. Such a shift would not negate the importance of protection but rather balance protection with empowerment, acknowledging children as active contributors to their own health and well-being. This also entails critically examining adultist assumptions embedded in policy, where authority and expertise are predominantly located with adults. Such a shift requires reorienting Nordic public health policy away from its dominant focus on prevention towards health promotion and empowerment principles, recognizing children as competent actors in the present, not merely investments for the future.

## Data Availability

Data derived from a source in the public domain. The data underlying this article are available at https://www.folkhalsomyndigheten.se/publikationer-och-material/publikationsarkiv/p/pa-vag-mot-en-god-och-jamlik-halsa-stodstruktur-for-det-statliga-folkhalsoarbetet/ and https://www.regjeringen.no/no/dokumenter/meld.-st.-15-20222023/id2969572/.
